# Repositioning Natural Products in Modern Drug Discovery: Technological Innovation, Systems Pharmacology, and Pathological Validation

**DOI:** 10.3390/ijms27104330

**Published:** 2026-05-13

**Authors:** Kazuhiko Nakadate, Nozomi Ito, Kiyoharu Kawakami

**Affiliations:** Department of Functional Morphology, Meiji Pharmaceutical University, 2-522-1 Noshio, Kiyose 204-8588, Tokyo, Japan; m256206@std.my-pharm.ac.jp (N.I.); k-kawakami@my-pharm.ac.jp (K.K.)

**Keywords:** natural products, systems pharmacology, molecular networks, omics integration, experimental pathology, drug discovery

## Abstract

Natural products have historically been integral to pharmacotherapy, attributed to their remarkable structural diversity and evolutionary refinement for biological interactions. Nonetheless, traditional natural product-based drug discovery has faced challenges such as mechanistic ambiguity, scalability limitations, and inadequate translational predictability. Concurrently, reductionist single-target approaches have been insufficient for addressing complex diseases characterized by network-level dysregulations. Recent advancements in analytical chemistry, genomics, and data-driven methodologies have rejuvenated natural product research by facilitating rapid structural elucidation, systematic exploration of biosynthetic diversity, and rational prioritization of bioactive compounds. Notably, many natural products exhibit multitarget effects that necessitate interpretation beyond isolated molecular interactions. Systems pharmacology offers a quantitative framework to analyze such network-level perturbations by integrating omics data, computational modeling, and experimental validation. However, molecular and computational predictions alone do not suffice to establish therapeutic relevance. Experimental pathology, encompassing histopathology, immunohistochemistry, spatial analysis, and ultrastructural evaluation, remains essential for validating efficacy and safety at tissue and organ levels. This review synthesizes technological innovation, systems pharmacology, and pathological validation to reposition natural products as mechanistically grounded and translationally robust resources for contemporary drug discovery.

## 1. Introduction

### 1.1. Repositioning Natural Products in Modern Drug Discovery

Natural products have been instrumental in the development of contemporary pharmacotherapy, contributing either directly or indirectly to a significant proportion of clinically approved pharmaceuticals [1]. Notable therapeutic categories—including antibiotics, anticancer agents, immunosuppressants, antiparasitic drugs, and cardiovascular medications—originate from natural sources or their derivatives [2]. Representative examples include paclitaxel as an anticancer agent, artemisinin as an antimalarial drug, and cyclosporine as an immunosuppressant, all of which highlight the clinical success and translational potential of natural products. These compounds illustrate how chemical entities, shaped by evolutionary pressures, can interact with biological systems in a highly selective yet functionally versatile manner [2]. Importantly, natural products are disproportionately represented among first-in-class drugs, underscoring their unique ability to access novel biological spaces and address unmet medical needs that remain resistant to conventional synthetic approaches [3,4].

Despite the historical success of natural product-based drug discovery, interest in this approach waned during the late twentieth century [5]. This decline coincided with the emergence of combinatorial chemistry, high-throughput screening platforms, and reductionist, target-based discovery paradigms [6]. These methodologies promised rapidity, scalability, and industrial efficiency; however, they often resulted in libraries of structurally simplified compounds with limited chemical diversity and modest biological relevance [4]. Consequently, many candidates demonstrated suboptimal efficacy, poor pharmacokinetic properties, or unexpected toxicity in later stages of development [7]. Concurrently, traditional natural product research encountered practical and conceptual challenges, including low compound yields, variability in biological source materials, difficulties in large-scale production, and limited tools for mechanistic elucidation [8].

Over the past two decades, the limitations of single-target paradigms have become increasingly apparent, particularly in the context of complex, multifactorial diseases such as cancer, metabolic disorders, immune-mediated conditions, and neurodegenerative diseases [9]. In these contexts, disease progression results not from the dysfunction of isolated molecular targets but from disturbances in interconnected signaling, metabolic, and regulatory networks [10]. Therapeutic interventions that focus narrowly on single nodes within these networks often fail to achieve sustained efficacy or are compromised by compensatory mechanisms and acquired resistance [11]. Network medicine studies have demonstrated that disease phenotypes arise from perturbations in interconnected molecular networks rather than isolated targets, highlighting limitations of reductionist approaches and supporting the need for multitarget strategies. For example, network analyses have revealed that disease-associated genes tend to cluster within specific interactome modules, and perturbation of these modules rather than single targets better explains disease phenotypes.

The paradigm shift has stimulated renewed interest in natural products as privileged chemical entities for network-level modulation [9]. Due to their inherent structural complexity, stereochemical richness, and functional group diversity, natural products often engage multiple molecular targets simultaneously [12]. Such pleiotropic activity, once considered a liability, is now increasingly recognized as a potential advantage for restoring network homeostasis in complex disease states [13]. Notably, contemporary advances have addressed many of the historical barriers associated with natural product research. High-resolution analytical chemistry enables rapid structural elucidation from minute quantities of material, while genome sequencing and mining technologies reveal vast, previously inaccessible biosynthetic potential [14]. Concurrently, computational biology and artificial intelligence facilitate rational prioritization and mechanistic hypothesis generation [15]. This rapidly evolving field is driven by advances in high-throughput omics technologies, genome mining, and artificial intelligence, which collectively improve the identification, prioritization, and mechanistic interpretation of bioactive natural products.

In the context of this dynamic field, systems pharmacology has become an essential framework for quantitatively and integratively interpreting the actions of multitarget drugs [16]. By integrating omics data, network modeling, and experimental validation, systems pharmacology facilitates a systematic examination of how natural products disrupt biological networks at various scales. Nonetheless, predictions at the network level necessitate rigorous validation to confirm their translational significance. In this context, experimental pathology plays a pivotal role by providing direct evidence that molecular and cellular disruptions lead to significant outcomes at the tissue and organ levels. Techniques such as histopathology, immunohistochemistry, and ultrastructural analyses capture comprehensive biological responses, thereby bridging the gap between mechanistic understanding and therapeutic efficacy [17,18]. These advances enable the prioritization of candidates based on predicted network impact, rather than single-target affinity alone.

**Figure 1:**
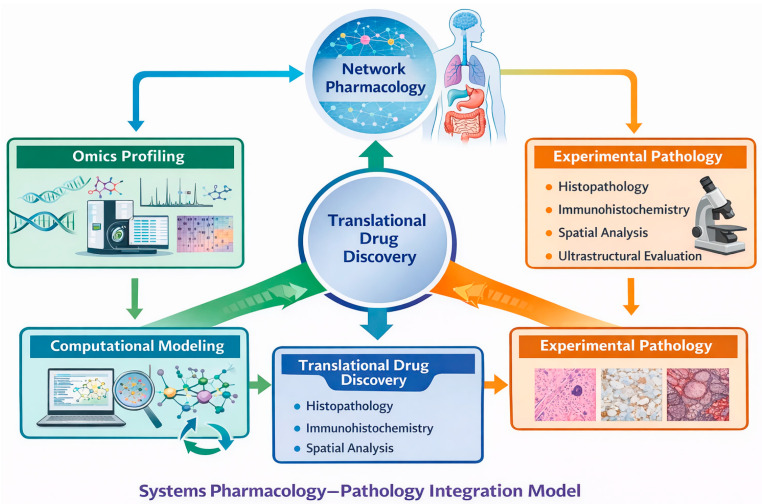
Integrative framework for translational drug discovery of bioactive natural products. Recent technological innovations—including analytical chemistry, genomics and metabolomics, and artificial intelligence (AI)—have significantly expanded access to bioactive natural products. These advances facilitate the identification and characterization of complex chemical entities. Systems pharmacology enables the integration of multiscale biological data through network analysis, omics integration, and computational modeling, thereby elucidating multitarget mechanisms of action. Concurrently, pathological validation—including histopathology, immunohistochemistry, spatial analysis, and ultrastructural studies—provides critical evidence linking molecular perturbations to tissue- and organ-level outcomes. The convergence of these approaches supports preclinical and translational assessment, ultimately driving the development of innovative therapies for complex diseases. The three curved arrows represent an iterative cycle of discovery, computational prediction, experimental validation, and feedback refinement. Histopathology is presented as a representative validation modality, complementing molecular and computational approaches. While histopathology is highlighted as a representative modality, other validation approaches, including molecular and functional assays, are also integral depending on the biological context.

In this review we consolidate recent advancements in analytical chemistry, genome science, systems pharmacology, and experimental pathology to redefine the role of natural products in contemporary drug discovery. By emphasizing mechanistic rigor and pathological validation, we propose a framework in which natural products are repositioned not as empirical remedies but as scientifically grounded and translationally robust resources for modern pharmacotherapy. Accordingly, this review is organized to provide a structured and integrative perspective on the contemporary repositioning of natural products in drug discovery. Section 2 outlines the historical evolution of natural product research and critically examines the factors that contributed to its temporary decline, establishing the conceptual context for its current resurgence. Section 3 discusses recent technological advances—including analytical chemistry, genome mining, and data-driven approaches—that have revitalized mechanistic discovery and expanded access to chemical diversity. Section 4 introduces systems pharmacology as a unifying framework for understanding multitarget actions of natural products and for interpreting network-level perturbations underlying therapeutic efficacy and toxicity. Section 5 focuses on experimental pathology as a critical translational bridge, detailing how histopathological, immunohistochemical, and ultrastructural analyses validate molecular and systems-level findings at the tissue and organ levels. Section 6 presents representative case studies that exemplify the integration of technological innovation, systems pharmacology, and pathological validation in natural product research. Finally, Section 7 discuss current challenges, future perspectives, and broader implications for the rational incorporation of natural products into modern drug discovery pipelines. To provide a comprehensive conceptual framework, Figure 1 summarizes how advances in analytical chemistry, omics technologies, and artificial intelligence integrate with systems pharmacology and pathological validation to enable the translational development of bioactive natural products for complex diseases.

In this review, ‘repositioning’ refers not only to the classical concept of drug repurposing but also to the systematic recontextualization of natural products within modern frameworks such as systems pharmacology, omics integration, and data-driven prioritization. The objective of this review is to propose an integrative framework that combines technological innovation, systems pharmacology, and pathological validation to reposition natural products as mechanistically grounded and translationally actionable therapeutic resources.

### 1.2. Literature Search Strategy and Methodological Considerations

This narrative review was conducted through a structured literature search designed to identify representative and conceptually relevant studies related to natural product-based drug discovery, systems pharmacology, and pathological validation. Literature searches were primarily performed using PubMed and Web of Science databases. The search strategy incorporated combinations of keywords and Medical Subject Headings (MeSH) terms, including “natural products,” “drug discovery,” “systems pharmacology,” “network pharmacology,” “metabolomics,” “genome mining,” “artificial intelligence,” “histopathology,” “immunohistochemistry,” and “translational pathology,” combined using Boolean operators such as AND/OR.

Priority was given to peer-reviewed articles published in English, particularly studies published between 2000 and 2025 that reflected recent technological and conceptual advances in the field. Seminal earlier publications were additionally included when considered historically or mechanistically important. The review preferentially included experimental studies involving in vitro, in vivo, translational, and systems-level analyses, as well as high-impact review articles relevant to the conceptual framework of this manuscript. Articles lacking sufficient methodological clarity or direct relevance to the scope of this review were not prioritized. As this article is a narrative review, formal systematic review protocols such as PRISMA were not applied. Representative landmark studies were selectively incorporated to provide historical and mechanistic context.

## 2. Natural Products as Privileged Chemical Scaffolds

Natural products represent a unique and highly valuable sector of chemical space that is not easily accessible through conventional synthetic methodologies [19]. Their molecular structures are typically characterized by high stereochemical density, rigid and well-defined three-dimensional frameworks, and a rich diversity of functional groups [20]. These structural attributes facilitate precise and stable interactions with biological macromolecules, including enzymes, receptors, and nucleic acids [21]. Importantly, such complexity is not arbitrary but reflects evolutionary selection pressures that favor molecular recognition and bioactivity within biological systems [22]. This evolutionary optimization underpins the concept of natural products as privileged chemical scaffolds capable of engaging multiple biological targets with high specificity and functional relevance [23].

Comparative analyses of chemical libraries and drug databases have consistently indicated that natural products and their derivatives are disproportionately represented among clinically approved pharmaceuticals [24]. This enrichment is particularly evident in therapeutic domains characterized by complex and dynamic signaling networks, such as oncology, immunology, and infectious diseases [9]. These findings suggest that molecular complexity, conformational rigidity, and functional group diversity provide distinct advantages for modulating multifaceted biological processes. In contrast, many synthetic compound libraries prioritize structural simplicity, planarity, and synthetic tractability [25]. While these features facilitate rapid synthesis and screening, they may limit the capacity of these compounds to engage complex protein surfaces or exert sustained effects within interconnected biological networks [26]. Specifically, this integration enables the interpretation of whether molecular-level changes translate into functionally meaningful tissue responses.

Natural products, beyond their inherent bioactivity, serve as highly adaptable foundations for the optimization processes in medicinal chemistry [9]. Historically, the semi-synthetic modification of natural scaffolds has resulted in numerous clinically successful agents characterized by enhanced potency, improved pharmacokinetic properties, and reduced toxicity [24]. Strategic modifications, including functional group alterations, stereochemical refinement, and scaffold simplification, facilitate the fine-tuning of target selectivity and drug-like properties while preserving the core structural features responsible for biological activity [27,28]. In this regard, natural products should be perceived not as finalized therapeutic agents but as versatile templates amenable to rational optimization [29].

Moreover, the notion of privileged scaffolds extends beyond individual targets to include modulation at the network level [30]. The capacity of natural products to engage with multiple components of biological systems may facilitate balanced modulation of signaling pathways, thereby diminishing the probability of resistance or compensatory activation [31]. This characteristic is closely aligned with contemporary perspectives on disease biology, which prioritize network dysfunction over isolated molecular defects [10]. When integrated with systems pharmacology and experimental pathology, privileged natural scaffolds offer a robust foundation for the development of mechanistically informed and translationally effective therapeutics [32,33].

While the conceptual significance of natural products as privileged chemical scaffolds is well recognized, their effective integration into contemporary drug discovery has historically been limited by technical and methodological constraints [28]. Challenges associated with compound identification, structural elucidation, scalability, and mechanistic interpretation have impeded the systematic exploitation of their full potential [9]. However, recent advancements in analytical chemistry, genome science, computational methods, and data-driven discovery have fundamentally transformed this landscape [34]. These technological innovations now facilitate comprehensive characterization, rational prioritization, and mechanistic interrogation of natural products with a level of precision and throughput previously unattainable [35,36]. The following section examines how these technological advances have revitalized natural product research and facilitated its convergence with contemporary drug discovery paradigms [37].

## 3. Technological Advances Driving Mechanistic Discovery

### 3.1. Advanced Analytical Chemistry and Structural Elucidation

The rapid advancement of analytical chemistry has significantly transformed both the feasibility and scientific rigor of natural product research [38]. High-resolution mass spectrometry (HRMS), encompassing time-of-flight and Orbitrap-based platforms, facilitates precise mass determination, isotope pattern analysis, and high-quality fragmentation profiling from minimal quantities of complex biological extracts [39,40]. These capabilities have substantially reduced the material requirements traditionally associated with the isolation and characterization of natural products, thereby expediting early-stage discovery [14,41]. Complementary techniques, including optical rotation and chiroptical spectroscopy, can further support stereochemical assignment and optical purity evaluation.

When combined with multidimensional nuclear magnetic resonance (NMR) spectroscopy—which includes two-dimensional and three-dimensional experiments such as COSY, HSQC, HMBC, and NOESY—HRMS enables precise structural elucidation, even for highly complex and stereochemically intricate molecules [14,42]. The integration of mass spectrometry and NMR data is particularly crucial for determining stereochemistry, conformational preferences, and subtle structural variants that often influence biological activity [43]. Collectively, these advancements have transformed structure elucidation from a rate-limiting bottleneck into a systematic and reproducible process [44].

Metabolomics-based strategies extend beyond the identification of individual compounds, offering a comprehensive, systems-level perspective on the chemical diversity present within biological samples [45]. By integrating chromatographic separation with HRMS and computational annotation, metabolomics facilitates the correlation of chemical features with biological phenotypes across various experimental conditions [46]. Feature-based molecular networking further augments this methodology by organizing structurally related metabolites into visually interpretable clusters, thereby aiding in the dereplication of known compounds and prioritizing chemically novel and biologically significant scaffolds [47,48]. Significantly, these analytical advancements have redirected natural product research from exhaustive, compound-centric isolation towards a hypothesis-driven approach in chemical biology [28]. Researchers are now able to concentrate on chemically and biologically informative subsets of metabolites, guided by phenotypic outcomes or mechanistic hypotheses [46]. This paradigm shift enhances efficiency, reproducibility, and mechanistic clarity, thereby aligning natural product research more closely with contemporary drug discovery workflows [9,49].

Despite significant advancements, the field of analytical chemistry-driven natural product discovery continues to encounter several limitations that require careful consideration [9]. The structural elucidation of highly complex molecules, particularly those present in ultra-low abundance, remains challenging even with the most advanced instrumentation [14]. Ambiguities in stereochemical assignment, conformational heterogeneity, and the presence of closely related analogs can complicate data interpretation and necessitate extensive validation [43]. Furthermore, metabolomics-based approaches often yield a large number of detected features, many of which lack confident structural annotation due to incomplete spectral databases [50]. Another challenge lies in correlating analytical data with biological relevance. While chemical profiling can efficiently characterize molecular diversity, it does not inherently distinguish bioactive compounds from chemically abundant but biologically inert metabolites [28]. Consequently, analytical advancements must be coupled with robust bioassays, phenotypic screens, and mechanistic hypotheses to ensure translational relevance [51]. Addressing these challenges requires the continued integration of analytical chemistry with computational annotation, experimental validation, and biological context [35].

### 3.2. Genome Mining and Biosynthetic Pathway Discovery

Genome mining has become a transformative approach for uncovering the extensive, previously concealed diversity of natural products encoded within microbial, plant, and marine genomes [47]. The advent of next-generation sequencing technologies, in conjunction with advanced bioinformatic tools, facilitates the systematic identification and annotation of biosynthetic gene clusters responsible for the synthesis of polyketides, nonribosomal peptides, terpenoids, alkaloids, and hybrid natural products. These gene clusters often remain transcriptionally inactive under standard laboratory conditions, yet they constitute significant reservoirs of unexplored chemical diversity [52,53].

Genome mining is crucial for elucidating not only the chemical diversity of natural products but also the biosynthetic principles that govern their assembly [34,54,55]. A comprehensive understanding of enzymatic structures, substrate specificity, and modular organization facilitates the rational modification of biosynthetic pathways through genetic engineering [37,56]. Such modifications include pathway activation, domain swapping, and precursor feeding strategies, which collectively enhance chemical diversity beyond what is naturally observed [57].

Metagenomic methodologies expand genome mining to include organisms that cannot be cultivated, thereby uncovering biosynthetic potential within diverse and intricate ecological niches [58,59]. These approaches circumvent long-standing cultivation challenges and substantially enlarge the accessible chemical space [60]. Additionally, heterologous expression systems facilitate the controlled production of target compounds, effectively addressing issues related to scalability, reproducibility, and supply [60,61]. By correlating genetic information with chemical output, genome mining establishes a direct mechanistic link between genotype and bioactive phenotype.

While genome mining has significantly enhanced access to biosynthetic diversity, several challenges impede its full utilization [54]. Numerous biosynthetic gene clusters remain transcriptionally inactive or exhibit low expression levels under laboratory conditions, and activation strategies are not universally effective [62]. Moreover, bioinformatic predictions of gene cluster function do not consistently result in successful compound isolation, as factors such as post-translational regulation, precursor availability, and cellular context affect biosynthetic output [63]. Although heterologous expression is a powerful tool, it presents its own set of challenges, including host compatibility, pathway toxicity, and incomplete reconstruction of native biosynthetic environments [64]. Additionally, translating genetic information into pharmacologically relevant chemical diversity necessitates close collaboration among genomics, chemistry, and biology. Addressing these limitations requires iterative experimental design and highlights the need for integrative frameworks that link biosynthetic potential with functional biological outcomes [54].

### 3.3. Artificial Intelligence and Data-Driven Discovery

Artificial intelligence (AI) and machine learning have become increasingly essential in contemporary natural product research, particularly in the management and interpretation of the extensive datasets generated by analytical chemistry, genomics, and phenotypic screening [65]. Predictive models, trained on chemical structure, bioactivity, and omics data, facilitate in silico prioritization of candidate compounds, prediction of pharmacokinetic properties, and early assessment of toxicity liabilities [66]. These tools mitigate experimental attrition and concentrate resources on candidates with greater translational potential [15].

AI-driven methodologies significantly enhance structure–activity relationship (SAR) analysis and the virtual screening of chemical spaces inspired by natural products [67]. When combined with genome mining and metabolomics, machine learning models can predict biosynthetic potential, inform experimental design, and uncover previously unrecognized structure–function relationships [37]. These data-driven strategies facilitate hypothesis generation and allow for the iterative refinement of discovery pipelines [66]. Crucially, AI and computational models serve as complementary tools that augment mechanistic insight rather than replace experimental validation [68]. When integrated with systems pharmacology frameworks, AI-driven analyses aid in the interpretation of multitarget interactions and network perturbations induced by natural products. This integrative approach establishes a coherent foundation for linking chemical structure, biological mechanism, and pathological outcome, thereby preparing the groundwork for the systems-level analyses discussed in the subsequent section [15].

Despite increasing enthusiasm, AI-driven methodologies in natural product research are limited by issues related to data quality, interpretability, and generalizability [66]. Machine learning models inherently rely on the availability of well-annotated training datasets, which remain scarce for numerous classes of natural products [68]. Biases present in existing datasets may skew predictions towards well-studied chemical scaffolds, potentially neglecting novel or unconventional structures [69]. Furthermore, AI-generated predictions often lack mechanistic transparency, complicating the inference of causal relationships between chemical features and biological effects [67]. Without experimental validation, computational outputs risk being overinterpreted or misapplied [15]. Consequently, AI should be regarded as an enabling technology rather than a standalone solution, functioning most effectively when integrated with experimental systems pharmacology and pathological validation [70].

Recent advancements in analytical chemistry, genome mining, and data-driven discovery have significantly broadened access to the chemical space of natural products and enhanced the efficiency of mechanistic hypothesis generation [9]. Nevertheless, these technologies predominantly function at the molecular, pathway, or isolated biological process levels [71]. Given that natural products often exhibit pleiotropic effects across multiple targets, interpreting their therapeutic potential necessitates frameworks capable of integrating these diverse signals into coherent biological narratives. Systems pharmacology offers such a framework by facilitating quantitative and network-based analysis of multitarget drug actions [32]. The subsequent section examines how systems pharmacology bridges technological discovery with biological complexity, providing a unifying perspective for comprehending the mechanisms and translational implications of natural products [72].

Publicly available databases, including NPASS, TCMSP, and DrugBank, provide structured repositories of chemical, pharmacological, and target information that enable systematic prioritization of natural products. Recent studies integrating machine learning with these databases have demonstrated improved prediction of multitarget activity and pharmacokinetic properties. Recent multi-omics integration studies combining transcriptomics, proteomics, and metabolomics have demonstrated improved identification of network perturbations, although challenges in data harmonization and standardization remain.

## 4. Systems Pharmacology and Network-Based Drug Discovery

Systems pharmacology offers a conceptual and quantitative framework for comprehending drug action within the context of complex biological networks, as opposed to isolated molecular targets [32]. Unlike traditional reductionist paradigms, systems pharmacology acknowledges that therapeutic efficacy, off-target effects, and toxicity arise from the coordinated modulation of multiple molecular nodes and pathways operating across various biological scales [70]. This network-oriented perspective is particularly well-suited to the study of natural products, which often interact with diverse molecular targets through structurally complex and multifunctional chemical motifs [9,49].

Quantitative systems pharmacology synthesizes high-dimensional omics data, computational modeling, and experimental validation to elucidate network perturbations resulting from drug exposure. Transcriptomic, proteomic, and metabolomic datasets offer complementary perspectives on biological responses, while network inference and dynamic modeling facilitate the identification of critical hubs and feedback loops [73]. These methodologies support the prediction of synergistic or antagonistic drug interactions, inform the rational design of combination therapies, and enable the stratification of patient populations based on network-level biomarkers [74].

**Figure 2:**
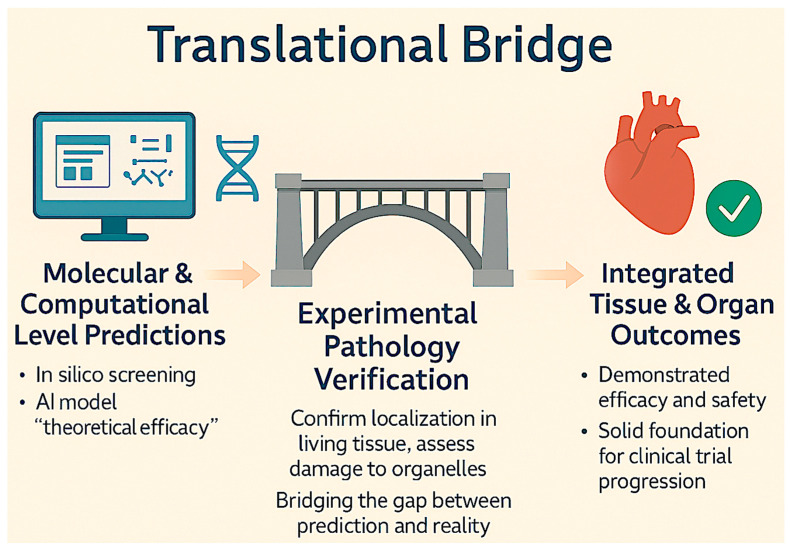
Experimental pathology as a translational bridge between molecular predictions and biological outcomes. Molecular and computational approaches, including in silico screening and artificial intelligence-based models, enable the prediction of potential therapeutic effects of natural products. However, these predictions require validation in biologically relevant systems. Experimental pathology provides this critical bridge by confirming molecular localization within living tissues, assessing subcellular and organelle-level alterations, and evaluating integrated tissue responses. Through histopathological, immunohistochemical, and ultrastructural analyses, experimental pathology links mechanistic predictions with functional biological outcomes. This integrative validation supports the assessment of both therapeutic efficacy and safety, thereby establishing a robust foundation for clinical translation. The figure has been optimized for clarity and readability, with simplified annotations and improved visual separation of key processes.

Systems pharmacology is inherently aligned with pathology-based evaluation, as both prioritize integrated biological outcomes over isolated molecular events. To establish translational relevance, network-level predictions necessitate validation at the tissue and organ levels [75]. Histopathological and ultrastructural analyses are crucial for confirming that network perturbations result in significant phenotypic outcomes, thereby grounding computational and molecular findings in biological reality [76]. This integration improves the linkage between molecular-level perturbations and tissue-level outcomes, thereby enhancing mechanistic interpretability and translational relevance. This convergence underscores the complementary roles of systems pharmacology and experimental pathology in modern drug discovery [32]. As shown in Figure 2, experimental pathology bridges the gap between in silico and molecular-level predictions and clinically relevant tissue and organ outcomes, providing essential validation of both efficacy and safety for translational drug development.

Despite its conceptual appeal, systems pharmacology encounters several methodological and practical challenges. The integration of heterogeneous omics datasets remains complex, as variations in data quality, temporal resolution, and biological context can complicate network inference [73]. Computational models frequently depend on simplifying assumptions that may not adequately capture nonlinear dynamics, cell-type specificity, or spatial heterogeneity within tissues [77]. Consequently, predicted network interactions may not consistently translate into experimentally observable outcomes [32]. Another challenge pertains to the interpretability of complex network models [66]. While systems-level analyses can identify correlated changes and candidate hubs, distinguishing causative drivers from downstream consequences remains challenging [10]. This issue is particularly pertinent for natural products, whose pleiotropic effects may obscure primary mechanisms of action [9]. Without careful experimental validation, there is a risk of overinterpreting network associations or attributing therapeutic relevance to epiphenomenal changes [15].

Translating insights from systems pharmacology into regulatory and clinical decision-making continues to present significant challenges. Network-based endpoints have not yet been routinely integrated into regulatory frameworks, and there is a lack of standardized criteria for their validation [78]. Overcoming these limitations necessitates a closer integration of computational modeling with experimental pathology, iterative refinement of models, and the development of consensus standards for systems-level evaluation [79]. Despite these limitations, systems pharmacology offers significant advantages by enabling the integration of heterogeneous datasets, capturing emergent network-level behaviors, and generating testable mechanistic hypotheses. Importantly, it should be viewed as complementary to reductionist approaches, which provide high-resolution insights into specific molecular interactions.

## 5. Pathological Validation as a Bridge to Translation

### 5.1. Limitations of Molecular and Cellular Assays

Molecular and cell-based assays are essential instruments in the early stages of drug discovery, facilitating the rapid identification of candidate compounds and the elucidation of proximal mechanisms of action [80]. Nevertheless, their predictive capacity for therapeutic efficacy and safety is inherently constrained [81]. Reductionist experimental systems frequently fail to replicate the architectural complexity, cellular heterogeneity, and dynamic microenvironments characteristic of intact tissues and organs [82]. As a result, molecular readouts may not accurately represent integrated physiological responses, compensatory signaling, or emergent toxicities that occur in vivo.

The limitations are particularly evident in the evaluation of natural products, which often demonstrate pleiotropic, context-dependent, and dose-dependent effects [9]. While multitarget modulation may seem beneficial at the molecular or cellular level, it can yield unexpected outcomes when integrated into tissue-level regulatory networks [70]. For instance, pathways that appear protective in isolated cell systems may exacerbate injury in the presence of immune–stromal interactions or altered extracellular matrix dynamics [83]. Consequently, reliance solely on molecular assays can result in both false-positive and false-negative evaluations of therapeutic potential [81].

Furthermore, frequently utilized immortalized cell lines often lack the differentiated phenotypes, metabolic capacities, and stress-response mechanisms that are characteristic of primary tissues [84]. This discrepancy further emphasizes the necessity for complementary in vivo and tissue-based evaluations to establish biological relevance and translational credibility [81].

### 5.2. Histopathological Evaluation of Efficacy and Toxicity

Histopathology continues to serve as a fundamental component of preclinical drug assessment, offering direct visual and quantitative evidence of disease modification and adverse effects. Through the systematic examination of tissue architecture, cellular morphology, and lesion distribution, histopathological analysis captures integrated biological outcomes that cannot be deduced from molecular markers alone [85]. Notably, pathology reflects the cumulative effects of molecular perturbations across temporal and biological scales [86].

In the realm of natural product research, histopathology plays a dual and essential role [9]. Firstly, it verifies that molecular or biochemical alterations result in significant tissue-level improvements, such as the reduction in inflammation, inhibition of fibrosis, maintenance of parenchymal structure, or prevention of cellular degeneration [87]. Secondly, it serves as a vital safety assessment tool, identifying organ-specific toxicities—including subtle degenerative, proliferative, or vascular lesions—that may not be detected through functional or biochemical assays [88].

Utilizing standardized pathological scoring systems, conducting blinded evaluations, and employing appropriate control comparisons significantly enhance the reproducibility and interpretability of research findings [89]. The integration of quantitative image analysis further bolsters objectivity by facilitating the correlation between pathological severity and molecular, pharmacokinetic, or pharmacodynamic parameters [90]. Such correlations are crucial for defining dose–response relationships and therapeutic windows [91].

### 5.3. Immunohistochemistry and Spatial Pathology

Immunohistochemistry (IHC) offers crucial spatial context by localizing molecular targets, signaling intermediates, and cellular phenotypes within intact tissue structures [92]. For natural products with multitarget actions, IHC enables the differentiation between direct target engagement and secondary or compensatory responses [9]. The capacity to localize pathway activation within specific cell populations—such as parenchymal cells, immune infiltrates, or stromal compartments—can elucidate mechanisms that remain unclear in homogenized tissue analyses.

Recent advancements in spatial pathology have significantly enhanced these capabilities [93]. Techniques such as multiplexed immunohistochemistry (IHC), imaging mass cytometry, and spatial transcriptomics facilitate the concurrent visualization of multiple molecular markers within specified tissue regions [94]. These methodologies elucidate intricate cellular interactions, gradients of signaling activity, and microenvironmental remodeling induced by therapeutic interventions [95]. Spatially resolved analyses are particularly beneficial in diseases characterized by localized inflammatory, fibrotic, or degenerative processes, where regional heterogeneity critically impacts disease progression and treatment response [96].

### 5.4. Ultrastructural Pathology and Subcellular Mechanisms

Ultrastructural pathology refers to the examination of cellular and subcellular architecture at nanometer-scale resolution using electron microscopy techniques, enabling direct visualization of organelles and intracellular processes. Ultrastructural pathology, facilitated by transmission and scanning electron microscopy, provides exceptional resolution for the assessment of subcellular changes [97]. Numerous natural products exert their biological effects by modulating organellar functions, such as mitochondrial metabolism, endoplasmic reticulum stress responses, lysosomal integrity, and autophagic flux [24]. Ultrastructural analysis allows for the direct visualization of these processes, thereby linking molecular signaling pathways with functional cellular outcomes [98]. For instance, the assessment of mitochondrial morphology can reveal early signs of metabolic dysfunction, oxidative damage, or cytoprotective adaptation [99]. Similarly, the evaluation of autophagosomes, lysosomes, and endoplasmic reticulum structures offers insights into cellular quality-control mechanisms and stress responses [100]. These analyses are particularly pertinent in research on neurodegeneration, metabolic diseases, and toxicological pathology, where subcellular changes often precede overt tissue damage [101].

Although ultrastructural approaches are technically challenging and characterized by low throughput, their capacity for mechanistic resolution renders them exceptionally valuable for validating hypotheses at the systems level and elucidating mechanisms that are not discernible through molecular assays alone [102].

### 5.5. Translational and Regulatory Perspectives

From a translational perspective, pathological endpoints are crucial in regulatory evaluation and risk assessment [103]. Regulatory agencies are increasingly prioritizing integrated safety assessment strategies that incorporate histopathological and ultrastructural findings alongside molecular, biochemical, and functional data [104]. For natural products, which are often perceived as inherently safe yet capable of complex biological activities, rigorous pathological validation is essential to establish scientific credibility and achieve regulatory acceptance [9,105].

The integration of pathology with systems pharmacology enhances translational confidence by correlating network-level modulation with specific tissue and organ outcomes [78]. This integrated approach facilitates rational dose selection, the identification of therapeutic windows, and the early prediction of adverse effects [79]. By grounding mechanistic and computational insights in pathological reality, experimental pathology serves as a crucial link between discovery science and clinical development [106].

## 6. Representative Case Studies of Natural Products

### 6.1. Tea Polyphenols as Multi-Scale Therapeutic Models

Tea-derived polyphenols, such as catechins and theaflavins, serve as exemplary natural products that have been extensively studied at molecular, cellular, tissue, and organismal levels [107,108]. These compounds demonstrate a wide range of bioactivities, influencing redox homeostasis, inflammatory signaling pathways, metabolic regulators, and mitochondrial function [109]. Their pleiotropic effects are indicative of their capacity to interact with multiple molecular targets and signaling pathways, rendering them highly suitable for systems-level investigation [110].

Mechanistic investigations have elucidated that tea polyphenols exert influence on critical regulatory nodes associated with oxidative stress responses, cytokine production, and energy metabolism [111]. These molecular effects result in cytoprotective and disease-modifying outcomes in experimental models of metabolic disorders, cardiovascular diseases, neurodegeneration, and inflammatory conditions [112]. Notably, the multitarget nature of polyphenols is consistent with contemporary perspectives on disease biology, which emphasize network dysregulation rather than isolated molecular defects [70].

Pathological analyses have critically validated these molecular findings. Histopathological assessments in models of metabolic and inflammatory diseases have consistently demonstrated reduced tissue injury, decreased infiltration of inflammatory cells, and preservation of organ architecture following polyphenol administration [9,113]. Ultrastructural analyses further reveal mitochondrial protection, enhanced membrane integrity, and stabilization of intracellular organelles, reinforcing mechanistic interpretations derived from molecular and biochemical assays [114]. Collectively, tea polyphenols exemplify how the integration of systems pharmacology and pathology can substantiate therapeutic potential across biological scales [72].

### 6.2. Alkaloids and Neuro–Immune Modulation

Representative examples include morphine, which modulates opioid receptors and immune signaling; berberine, which influences inflammatory and metabolic pathways; and nicotine, which affects cholinergic signaling and neuroinflammatory responses. Alkaloids represent a chemically diverse and pharmacologically potent class of natural compounds with significant effects on both the nervous and immune systems [115]. Many alkaloids interact with various receptor systems, ion channels, and intracellular signaling pathways, highlighting the importance of systems pharmacology in their evaluation. This multitarget activity underpins their therapeutic efficacy in neurological, psychiatric, and inflammatory disorders, while simultaneously presenting challenges related to safety and dose optimization [9].

Network-based analyses have demonstrated that alkaloids frequently modulate neuro-immune interactions, thereby affecting neurotransmission, neuroinflammation, and immune cell activation [116]. These effects cannot be sufficiently captured through single-target assays alone. Consequently, pathological evaluation has been crucial in defining safe and effective therapeutic windows for alkaloids [88]. Detailed histopathological and ultrastructural analyses facilitate the differentiation between neuroprotective modulation—such as the preservation of neuronal architecture and the suppression of inflammatory infiltration—and neurotoxic effects, including cellular degeneration or synaptic disruption [101]. By integrating molecular, network, and pathological data, studies on alkaloids exemplify how comprehensive evaluation frameworks can balance therapeutic potential with safety considerations, thereby informing translational development [117].

### 6.3. Microbial Secondary Metabolites and Network Modulation

Microbial secondary metabolites have been instrumental in the development of numerous clinically significant drugs, including antibiotics, immunosuppressants, and anticancer agents [24]. In addition to their direct antimicrobial or cytotoxic properties, recent research has underscored their ability to influence host–microbe interactions, immune signaling networks, and metabolic pathways [118]. These discoveries broaden the conceptual understanding of microbial natural products from specific target engagement to comprehensive network modulation.

Systems pharmacology approaches have been particularly valuable for elucidating these complex interactions, revealing how microbial metabolites influence host immune responses and tissue homeostasis. Pathological validation remains essential in this context, as modulation of immune and metabolic networks can produce both therapeutic benefits and adverse effects [103]. Integrative evaluation incorporating systems pharmacology and pathology has therefore been critical for optimizing therapeutic application and minimizing unintended consequences [78]. Microbial secondary metabolites thus exemplify the necessity of multiscale validation strategies in natural product research [9].

To systematically illustrate the relationship between natural product classes, their network-level targets, and pathology-based validation strategies, representative examples are summarized in Table 1. This table summarizes representative classes of natural products, their primary network-level targets, and key pathological validation endpoints. These examples highlight how distinct categories of natural compounds modulate specific biological networks and require tailored pathology-based evaluation strategies to confirm therapeutic efficacy and safety at the tissue and organ levels.

## 7. Challenges, Limitations, and Future Directions

Despite recent advancements and technological progress, substantial challenges persist in the translation of natural products into clinically viable therapeutics [9]. Variability in source materials, chemical complexity, and batch-to-batch inconsistency complicate efforts toward standardization and reproducibility. Furthermore, discrepancies between animal models and human disease limit translational predictability, particularly for complex, multifactorial conditions [81].

One significant challenge involves the integration of heterogeneous datasets produced by analytical chemistry, genomics, systems pharmacology, and pathology [73]. The harmonization of experimental design, data analysis, and reporting standards is crucial to ensure both interpretability and regulatory acceptance [119]. Additionally, the regulatory framework for natural products remains diverse, highlighting the necessity for rigorous, pathology-guided validation strategies [120].

Future advancements are anticipated to emerge from the convergence of natural product research with precision medicine, data science, and pathology-centered evaluation [121]. The development of standardized methodologies, enhanced disease models, and integrative analytical frameworks will be crucial for fully harnessing the therapeutic potential of natural compounds [122]. Such interdisciplinary approaches hold the promise of transforming natural products from empirically utilized agents into mechanistically grounded and clinically actionable therapeutics [105]. Future research should focus on establishing standardized integrative pipelines that combine computational prediction, multi-omics data, and pathology-based validation to enhance reproducibility and translational applicability.

## 8. Conclusions

Natural products continue to be a crucial yet underutilized resource for drug discovery within the framework of network medicine. Their inherent structural complexity, evolutionary refinement, and multitarget capabilities uniquely position them for the modulation of dysregulated biological networks that underlie complex diseases. By integrating technological advancements, systems pharmacology, and rigorous pathological validation, contemporary research can transcend historical limitations and unlock novel therapeutic opportunities.

This review highlights the significance of interdisciplinary approaches that connect molecular discovery with translational relevance. The integration centered on pathology serves as a crucial foundation for mechanistic understanding, ensuring that perturbations at the network level result in significant tissue and organ outcomes. Through such integrative frameworks, natural products can be repositioned as scientifically robust and translationally credible foundations for future pharmacotherapy. In this context, repositioning should not be interpreted solely as the identification of new therapeutic uses, but rather as a conceptual advancement in which natural products are redefined as network-modulating, mechanistically validated, and translationally actionable therapeutic entities.

## Figures and Tables

**Table 1:** Classes of Natural Products, Major Network Targets, and Pathological Validation Endpoints.

Class of Natural Products	Major Network Targets	Key Pathological Validation Endpoints
Tea polyphenols	Oxidative stress pathways, inflammatory cytokines, energy metabolism	Attenuation of tissue injury; visualization of mitochondrial protection
Alkaloids	Neurotransmitter receptors, ion channels, neuro–immune crosstalk	Histopathological evaluation to distinguish neuroprotective effects from neurotoxicity
Microbial secondary metabolites	Host–microbiota interactions, immune signaling networks	Histological screening for immunomodulation-associated adverse effects (toxicity)

Network targets refer to biological processes and pathways rather than single molecular entities, reflecting the systems-level actions of natural products.

## Data Availability

No new data were created or analyzed in this study. Data sharing is not applicable to this article.
